# Habitat-, age-, and sex-related alterations in oxidative stress biomarkers in the blood of mute swans (*Cygnus olor*) inhabiting pomeranian coastal areas (Northern Poland)

**DOI:** 10.1007/s11356-021-18393-3

**Published:** 2021-12-31

**Authors:** Natalia Kurhaluk, Halyna Tkachenko

**Affiliations:** Department of Biology, Institute of Biology and Earth Sciences, Pomeranian University in Słupsk, Arciszewski Str. 22b, 76-200 Słupsk, Poland

**Keywords:** Wintering population of mute swan (*Cygnus olor*), Oxidative stress biomarkers, Pollution, Al, Fe, Cu, Rh, Ru

## Abstract

The mute swan (*Cygnus olor*) can be considered a representative species of birds associated with the aquatic environment and responding very clearly to changes in the environment. Assuming that the condition of the mute swan population well reflects the state of the environment, this species was used in our research as a bioindicative species. Thus, the aim of our study was to elucidate the association between metal contents in soil samples collected from a habitat of mute swans and element contents in their feathers as well as the levels of biomarkers of lipid peroxidation, oxidatively modified proteins, and total antioxidant capacity in the blood of mute swans living in three agglomerations in coastal areas in the southern part of the Baltic Sea (Pomeranian region, northern Poland). We compared the effects of inhabitation, age, and sex on the ecophysiological accumulation of metals in three wintering populations of the mute swan from coastal areas of northern Poland, i.e., Słupsk, Gdynia, and Sopot. In Słupsk, the anthropogenic pressure was related predominantly to the level of Al and, to a lesser extent, to the content of Rh and Ru. We found maximum levels of lipid peroxidation biomarkers in the blood of the mute swans from Gdynia (38.20 ± 6.35 nmol MDA·mL^−1^). At the same time, maximum levels of aldehydic and ketonic derivatives of oxidatively modified proteins were noted in the blood of swans from Sopot compared to the values obtained in mute swans from Słupsk and Gdynia. This trend suggesting high levels of oxidative stress biomarkers was also confirmed by a decrease in the total antioxidant capacity in these groups.

## Introduction

The functioning of the environment of living organisms has an impact on various levels of ecosystem organization (Marchowski et al. 2018). The Baltic Sea is one of the largest brackish seas in the world; such properties make it particularly vulnerable to pollution and eutrophication (Dietz et al. 2021). The very long period of total water exchange in the Baltic Sea (25–30 years) is one of the factors making it one of the most polluted seas in the world (Sonne et al. 2020a). For this reason, ecotoxicological studies are being conducted to identify effective bioindicators while determining the level of pollution (Sonne et al. 2020b). This approach helps to assess the degree of xenobiotic impacts on the population and structure of aquatic and terrestrial coastal ecosystems (Binkowski et al. 2016).

The sandy beaches of the Baltic Sea receive large quantities of organic matter, for example, from seawater. Such accumulation of organic matter generates optimal conditions for the growth of many heterotrophic microorganisms, mainly bacteria (Zander and Reimer 2002; Sonne et al. 2020a,b). The beaches of Sopot, Gdynia, and Gdańsk are places of recreational activity. They are frequented by tourists, whose density in summer reaches 30 people per 100 m^2^, and even about 3,000 people can be present there daily (Węsławski et al. 2006).

The mute swan (*Cygnus olor*) living on the Baltic Sea beaches in Sopot and Gdynia is the largest representative of the geese subfamily in Poland. It is a territorial species but sometimes nests in colonies of up to several hundred pairs (England, Denmark). Mute swans stay in pairs only in the breeding season, whereas in winter they form flocks up to some hundreds of individuals. They feed mainly in the mornings and afternoons on shallows. They sometimes go ashore to feed (Włodarczyk and Wojciechowski 2001). Beyond the breeding season, they feed all day and not only on shallows. A high number of swans are regularly observed on fields sown with winter crops.

Mute swans live only in places where the environment is not severely transformed and birds are able to find rich feeding grounds that can assure their survival (Charmantier et al. 2006; Dolka et al. 2014). Analysis of the causes of changes in the distribution and swan population size on a regional scale provides valuable information on the threats to this species. Mute swans are a good object for indication research because it helps acquire knowledge of the environment and dependencies whilst shaping an active attitude toward the surrounding environment (Meissner et al. 2001).

The ringing of swans has shown that during cold winters more swans fly to wintering grounds in western (France, Netherlands, Denmark) and southern (Hungary, Croatia, and even Greece) Europe, while in Poland more birds come from the north and east. It has been estimated that around 330,000 mute swans spend the winter in Europe. In the last several years, 15,000–24,000 mute swans have been wintering in Poland. The largest populations (up to 3,800 birds in 1993) were observed in the Gulf of Gdańsk. The highest density of birds wintering on the Polish coast is observed in the Gulf of Gdańsk (> 10 birds/km^2^), Vistula Bay, Szczecin Bay, and the Szczecin coast (1–9 birds/km^2^) (Meissner et al. 2001).

Mute swans once represented typically migratory birds. Currently, up to 80% of these swans stay in Poland for the winter (Wieloch and Remisiewicz, 2001). Wintering and feeding swans, i.e., birds wintering in large urban agglomerations, are particularly useful bioindicators of the degree of environmental pollution. These large individuals are conspicuous and vulnerable to toxic substances, and their trophic chain is linked to the environment (Schummer et al., 2011). Furthermore, the birds remain natural components of ecosystems. They are therefore considered to be an early bioindicator of environmental stress and are of particular interest for ecotoxicological studies in natural conditions (Abdullah et al. 2015; Karimi et al. 2016).

The biomonitoring of elements in living organisms is carried out all over the world (Karimi et al., 2016). Birds are susceptible to environmental changes, which make them particularly important as indicators of environmental contamination, including metal concentrations (Tkachenko and Kurhaluk 2012; Tkachenko et al. 2021; Kurhaluk et al. 2021). The mute swan (*Cygnus olor* Gmelin, Anatidae), i.e., a common water bird of lowland freshwaters and coastal shallows, is an effective model system of environmental contamination (Beyer and Day 2004; Grúz et al. 2015, 2018, 2019).

Free radical-induced pathology has become the most common pathology in humans and animals in the late twentieth and early twenty-first century (Valko et al., 2016). Nowadays, there are increasing numbers of publications on oxidative stress as one of the most important pathogenic mechanisms of many disorders and diseases (Bjørklund et al., 2020). The assessment of oxidative stress biomarkers serves as a good tool for biomonitoring of animal welfare and their environmental living conditions. Birds living close to humans are often used as an effective marker of these processes (Karimi et al., 2016).

Oxidative stress is a phenomenon caused by an imbalance between the production and accumulation of oxygen reactive species (ROS) in cells and tissues (Pizzino et al., 2017). ROS are normally generated as by-products of oxygen metabolism, and they can play several physiological roles, e.g., as regulatory mediators in signaling processes, including differentiation, autophagy, metabolic adaptation, apoptosis, and immunity (Dröge 2002; Ikeda and Nagasaki 2018). Environmental stressors (UV, ionizing radiations, pollutants, and heavy metals) and xenobiotics (pesticides, antiblastic drugs, etc.) greatly contribute to an increase in ROS production, thereby causing an imbalance that leads to oxidative stress-induced damage to cells and tissues (Pizzino et al., 2017). Metals can cause oxidative stress by increasing ROS formation. Metal toxicity is related to their oxidative state and reactivity with other compounds (Koivula and Eeva, 2010). ROS can attack biomolecules directly, with consequent enhancement in membrane lipid peroxidation, protein oxidation, and DNA damage (Videla et al., 2003). ROS-mediated reactions lead to the formation of protein carbonyl derivatives, which serve as a marker of ROS-mediated protein damage or convert lipid and carbohydrate derivatives to compounds that react with functional groups on proteins (Stadtman and Berlett 1998). Excessive activation of lipid peroxidation chain processes leads to the accumulation of lipid peroxides, fatty acid radicals, ketones, aldehydes, and ketoacids in tissues, which in turn cause damage and an increase in cell membrane permeability as well as oxidative modification of structural proteins and enzymes (Johnson and Decker 2015).

Thus, we hypothesized that environmental contamination, including metal-induced pollution, can cause oxidative stress and reduce antioxidant capacity in the blood of mute swans. The goal of the current study was to elucidate the association between metal contents in soil samples collected from a habitat of these birds and element contents in their feathers as well as the levels of biomarkers of lipid peroxidation, oxidatively modified proteins, and total antioxidant capacity in the blood of mute swans living in three agglomerations in coastal areas in the southern part of the Baltic Sea (Pomeranian region, northern Poland).

## Materials and methods

These investigations were performed in accordance with the Guidelines of the European Council and the current laws in Poland. This study was approved by the General Directorate for Environmental Protection according to Permission DOP-oz. 6401.03.278.2012.km of Poland for Natalia Kurhaluk, Halyna Tkachenko, and Maria Wieloch.

### Study areas

**Fig. 1:**
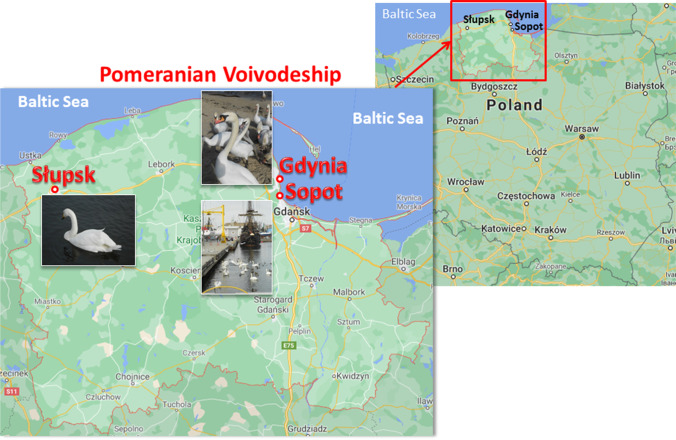
Study areas. Wintering population of the mute swan (Cygnus olor) in Słupsk, Gdynia, and Sopot (northern Poland)

The study was conducted in Słupsk and the Gulf of Gdańsk on the southern Baltic coast. The latter localities were the municipal beaches of two neighboring cities, Sopot and Gdynia, in Northern Poland (Fig. 1). In Słupsk, birds were collected from municipal Park lakes, where 12–15 mute swans lived during their non-breeding period. The municipal beaches of Sopot and Gdynia are inhabited by more than one hundred birds (Meissner et al. 2020).

The soil, feathers, and blood samples were collected between October and November in 2012–2013. Each bird was sampled only once. The juvenile birds from all habitats were below three years of age, and adult specimens were over three years old. The group in Słupsk comprised 10 male juveniles, 12 male adults, 10 female juveniles, and 9 female adults. In Gdynia, 12 male juveniles, 18 male adults, 14 female juveniles, and 12 female adults were analyzed. The group of birds from Sopot consisted of 10 male juveniles, 17 male adults, 14 female juveniles, and 13 female adults. In total, samples of feathers and blood were collected from 151 birds.

The mute swans were sexed by cloacal examination (Brown and Brown 2002), and their age was identified based on plumage characteristics and color of the bill (Baker 2016). As the majority of birds had been ringed previously as part of other projects, their history, i.e., the date and age at ringing, was known (data provided by the Polish Bird Ringing Centre).

We analyzed two age groups: juvenile birds (before the third year of life) and adults (older than three years), as the mute swan starts to breed not earlier than in the third or fourth year of life (Coleman and Coleman 2002). All necessary biometric measurements were taken according to a methodology or standard procedures used in bird ringing stations and developed for mute swan studies (Mathiasson 2005). The physiological condition of the birds was good, the body mass of the birds used in this study was 7.32–11.23 kg (females) and 7.92–11.96 kg (males), and no individual showed pathological conditions and diseases.

### Blood and feather samples

Blood samples were collected from the medial metatarsal vein in a 3.8% sodium citrate solution and blood collection tubes with K_3_-EDTA (Vacuette®). All swans were marked with a numbered ornithological ring and released on the same water body where they were captured. Blood was stored in tubes on ice until centrifugation at 3,000 rpm for 15 min. The plasma was removed. The erythrocyte suspension (one volume) was washed with five volumes of a saline solution three times and centrifuged at 3,000 rpm for 15 min.

Contour feathers (2–3) were taken from the rump without disturbing the birds. They were first washed three times with tap water, splashed with distilled water, and then cleaned in acetone (Battaglia et al. 2003) to exclude the presence of external pollutants (Goede and Bruin 1984). Thereafter, the samples were dried in an oven at 30 °C for 48 h. The feathers were dissected into small pieces, and the samples were weighed in grams and used for analysis.

### Soil samples

In Słupsk, 12 soil samples (each sample was analyzed in three replicates) were taken from Lake Park, where the mute swan population lives. In Sopot and Gdynia, 12 soil samples (analysis of each sample was carried out in three replications) were taken from a coastal area near the Baltic Sea where the birds receive food and water (100 m in width, 100 m in length of the coastal zone). The soil samples were collected from topsoil at the depth of 0–5 cm using a soil sampler. In each of the three sites (Słupsk, Sopot, and Gdynia), four sub-sites were established at the perimeter of the area, and three soil samples were collected from each of the four sub-sites. Twelve samples, i.e., one from each site, were transferred into polyethylene bags. All sampled materials were picked up with a plastic shovel and stored in plastic bags to avoid manual contamination and then taken to the laboratory for further analysis. The samples were air-dried for one week, ground to pass through a 200-mesh sieve, and transferred to polyethylene bags until analysis. Each soil sample was analyzed in three series. Between different reads, the soil sample was thoroughly mixed in the same bag. The results of three reads were averaged.

### Determination of element concentrations

The concentrations of chemical elements were analyzed in the feather and soil samples with an X-ray fluorescence (XRF) analyzer (model Sci Sps X-200 from Sci Sps, Inc.).

The XRF (X-ray fluorescence) analyzer generates an X-ray beam that can be used for irradiating the sample as we described earlier (Kurhaluk et al. 2021; Tkachenko et al 2021). The X-ray fluorescence hyperspectral data were processed using PyMca 5.1.3 (Solé et al. 2007) and Datamuncher (Alfeld and Janssens 2015) software. The device software uses either standard methods such as basic parameters for the spectra of the given elements (we used this method in our measurements) or user-generated empirical calibration curves to relate the X-ray spectrum to the element concentrations.

### Biochemical assays

Blood and plasma were used for the determination of the levels of 2-Thiobarbituric acid reactive substances (TBARS), oxidatively modified proteins (OMP), and total antioxidant capacity (TAC). All reagents used in this study were purchased from Sigma-Aldrich (Sigma-Aldrich Sp. z o.o., Poznan, Poland) and Avantor Performance Materials Poland S.A. (Gliwice, Poland). The reagents were freshly prepared. All other reagents used were of analytical reagent grade. The specific assay conditions are presented below. Each sample was analyzed in duplicate.

### 2-Thiobarbituric acid reactive substances (TBARS) assay

The level of lipid peroxidation was determined by quantifying the concentration of 2-thiobarbituric acid reacting substances (TBARS) using the Kamyshnikov (2004) method for determination of the concentration of malonic dialdehyde (MDA). This method is based on the degradation of the lipid peroxidation product, MDA, with 2-thiobarbituric acid (TBA) at high temperature and acidity to generate a colored adduct, which is then measured spectrophotometrically. The nmol of MDA per mg protein was calculated using 1.56·10^5^ mM^−1^ cm^−1^ as the extinction coefficient.

### Protein carbonyl derivative assay

The assay of the carbonyl derivative content in oxidatively modified proteins (OMP) was performed based on the spectrophotometric measurement of aldehydic (AD) and ketonic derivatives (KD) in the tissue samples. The rate of protein oxidative destruction was estimated from the reaction of the resultant carbonyl derivatives of the amino acid reaction with 2,4-dinitrophenylhydrazine (DNFH) as described by Levine et al. (1990) and modified by Dubinina et al. (1995). DNFH was used for determination of the carbonyl content in soluble and insoluble proteins. Carbonyl groups were determined spectrophotometrically from the difference in absorbance at 370 nm (aldehydic derivatives, OMP_370_) and 430 nm (ketonic derivatives, OMP_430_).

### Total antioxidant capacity assay

The TAC level in the samples was estimated by measuring the TBARS level following Tween-80 oxidation. The level was determined spectrophotometrically at 532 nm, as in Galaktionova and co-workers (1998). The sample inhibited the Fe^2+^/ascorbate-induced oxidation of Tween-80, which resulted in reduced TBARS levels. The level of TAC in the sample (%) was calculated for the absorbance of the blank.

### Statistical analysis

The basic statistical analysis (significance of regression slopes, analysis of variance for significance between the habitats, age, and sex of mute swans for metals and oxidative stress biomarkers; distribution testing) was performed using the STATISTICA 13.3 package (TIBCO Software Inc.). The results were expressed as mean ± S.D. The arithmetic means of the data on oxidative stress in the blood were estimated using MANOVA. The data were tested for homogeneity and normality (Kolmogorov–Smirnov test). Firstly, significant differences among the means were detected using a multiple range test at min. P < 0.05. The correlation of parametric values was based on Pearson’s regression analysis using the multiple regression module. To illustrate the simple linear regression model in the statistical analysis, linkage models for the oxidative stress variables were conducted for each of the dependent variables (localization, sex, age). For this purpose, the regression of the dependent variable was summarized using multiple R, multiple R^2^, and multiple adjusted R^2^-values, with values of the F and the P level to confirm the statistical significance of the linear model. The levels and significance of differences in the levels of oxidative stress biomarkers were assessed with the F test and Bonferroni post hoc test (Zar 1999; Stanisz 2006, 2007).

## Results

### Metals

In our study, the analysis of variance for the three areas showed that the level of the following elements in the soil differed significantly: Al (F_2,11_ = 81.99, p = 0.000), Si (F_2,11_ = 179.22, p = 0.000), Ti (F_2,11_ = 74.32, p = 0.000), Mn (F_2,11_ = 22.52, p = 0.000), Fe (F_2,11_ = 150.20, p = 0.000), Cu (F_2,11_ = 111.66, p = 0.000), Zn (F_2,11_ = 20.97, p = 0.000), Zr (F_2,11_ = 4.55, p = 0.018), Rh (F_2,11_ = 96.81, p = 0.000), and Ru (F_2,11_ = 40.90, p = 0.000). There were no statistically significant differences in the Ni, Pb, and Pd values in the soil. In the soil samples (g/kg) from Słupsk, the levels of Al, Si, Ti, Mn, Fe, Cu, and Zn were statistically significantly different (p = 0.000) compared to the values in Gdynia and Sopot. The level of Rh and Ru differed statistically significantly in the three studied areas. The level of Zr was statistically significantly different only in the samples from Gdynia and Sopot (data not shown). Therefore, the analysis of the metal contents in the soil samples collected from the mute swan habitats showed different results. We suggested that there were alterations in their bioaccumulation in the organism of the birds caused by pronounced migration processes.

The next step of our investigation was to compare the results of metal contents in soil with those in the feathers (mg/kg) of mute swans living in the three localizations and differing in their age and sex. Then, we divided the study groups according to these criteria and carried out multivariate statistical analysis (MANOVA) to assess the influence of these factors on the bioaccumulation of elements in these animals. The dependencies between different levels of oxidative stress biomarkers in the blood of mute swans have determined our approach to the analysis, namely to determine differences in metal contents in the feathers, caused by the localization and type of urban agglomerations (large, small, remote from places visited by tourists, etc.), age and sex of birds, as well as the combination of these factors important for bird habitats. Based on the statistical analysis with sigma-restricted parameterization of the effective hypothesis decomposition, it was shown that the highest significant dependencies of the analyzed main effects regarding the distribution of the metals in feathers were the environmental impact (F = 44.73, p = 0.000) and its combination with the other main factors, i.e., age and sex (F = 32.20, p = 0.000 and F = 23.12, p = 0.000, respectively) in the holistic statistical model. The results indicated an association between the habitat and the levels of metals in the samples; the age and sex of the birds were involved in these processes as well (F = 28.0, p = 0.000; F = 25.96, p = 0.000, respectively).

Metal levels in feathers reflect the levels in food during the feather grows, i.e., during the grows of young birds, or during the moult period of adult birds. X-ray fluorescence hyperspectral analysis used in our work showed that distribution of metal (mg/kg) deviated significantly in the Al, Zn, Rh, Cu, and Ru. The samples in the group from Słupsk differed in the Al content, and those collected in Gdynia exhibited differences in the Ru (0.06 ± 0.05 mg/kg in juveniles females’ group) value. Samples from birds inhabiting the Sopot coastal areas had statistically different Ru (0.04 ± 0.05 mg/kg in adult’s male birds) and Cu (0.01 ± 0.01 mg/kg in adult females’ group) levels. The content of Al in the feathers of birds from the three study sites in the groups (juveniles and adult males, and juveniles and adult females) was similar (from 99.81 ± 0.05 to 99.72 ± 10.19 mg/kg), but we detected the lowest levels of this element in adult females from Słupsk (66.51 ± 49.83 mg/kg).

We used the SS (sum of squares) test as a statistical tool that is often used to identify the dispersion of data, as well as how well the data can fit the model in regression. We found in three-way ANOVA for our whole statistical model the increased levels of Al, Cu, and Ru in the feathers of birds from all groups, which indicated effective bioaccumulation processes in the wintering population of the mute swan. These results determined the next step of our study.

### Biomarkers of oxidative stress

The results of the biochemical studies of oxidative stress biomarkers showed the level of end products of lipid peroxidation processes, such as TBARS products in the blood and plasma of the population of wintering mute swans living in northern Poland (Table 1). ANOVA tests showed statistically significant differences in all the analyzed parameters in the group of juvenile and adult birds. As we expected, the highest values of the significant differences were related to the aldehydic and ketonic derivatives of oxidatively modified derivatives in the plasma of the juvenile birds as well as the aldehydic derivatives of OMP and total antioxidant capacity in the blood of the adult animals (Table 1).

**Table 1:** Analysis of variance of oxidative stress biomarkers with a two-way analysis of the age factor (juveniles, adults) in wintering mute swan populations from three coastal areas of the Pomeranian region (northern Poland)

Factor	Parameters	F	P
Juveniles	TBARS, blood	11.08	0.000
	TBARS, plasma	8.51	0.000
	Aldehydic derivatives of OMP, plasma	21.77	0.000
	Ketonic derivatives of OMP, plasma	113.04	0.000
	TAC, blood	2.41	0.046
Adults	TBARS, blood	3.61	0.006
	TBARS, plasma	7.68	0.000
	Aldehydic derivatives of OMP, plasma	45.37	0.000
	Ketonic derivatives of OMP, plasma	8.08	0.000
	TAC, blood	18.55	0.000

Based on these findings, we estimated the potential impact of the sex of the birds on levels of oxidative stress biomarkers. Table 2 shows the ANOVA test data in the groups of males and females of the mute swans inhabiting the three locations. In addition, the ANOVA analysis of oxidative stress biomarkers showed statistical significance except for TBARS and TAC values in the blood of the male birds. The level of oxidative stress biomarkers was associated with an increase in both aldehydic and ketonic derivatives of OMP in the plasma of male birds and aldehydic derivatives and TAC levels in the female group.

**Table 2:** Analysis of variance of oxidative stress biomarkers with a two-way analysis of the sex factor (males, females) in wintering mute swan populations from three coastal areas of the Pomeranian region (northern Poland)

Factor	Parameters	F	P
Males	TBARS, blood	2.11	0.073
	TBARS, plasma	7.25	0.000
	Aldehydic derivatives of OMP, plasma	27.84	0.000
	Ketonic derivatives of OMP, plasma	172.42	0.000
	TAC, blood	1.75	0.133
Females	TBARS, blood	7.67	0.006
	TBARS, plasma	8.50	0.000
	Aldehydic derivatives of OMP, plasma	52.84	0.000
	Ketonic derivatives of OMP, plasma	4.73	0.001
	TAC, blood	57.30	0.000

Table 3 shows the ANOVA results demonstrating significant statistical relationships between the biomarkers of oxidative stress in the blood and plasma of the mute swans from the three localizations. As we expected, the results of the ANOVA statistical tests demonstrated the highest levels of aldehydic and ketonic derivatives of OMP in the blood of mute swans from Słupsk. Similar high results were obtained in the analysis of the birds from Sopot. Furthermore, we found that the differences in the levels of oxidatively modified proteins were caused by the type of environmental contamination, as shown in this study by the analysis of the soil and feathers.

**Table 3:** Analysis of variance of oxidative stress biomarkers with a three-way analysis of the habitat factor (Słupsk, Gdynia, Sopot) in wintering mute swan populations from three coastal areas of the Pomeranian region (northern Poland)

Factor	Parameters	F	P
Słupsk	TBARS, blood	6.90	0.000
	TBARS, plasma	2.97	0.044
	Aldehydic derivatives of OMP, plasma	176.61	0.000
	Ketonic derivatives of OMP, plasma	238.26	0.000
	TAC, blood	73.82	0.000
Gdynia	TBARS, blood	12.02	0.006
	TBARS, plasma	14.18	0.000
	Aldehydic derivatives of OMP, plasma	16.83	0.000
	Ketonic derivatives of OMP, plasma	3.59	0.019
	TAC, blood	4.46	0.007
Sopot	TBARS, blood	4.98	0.004
	TBARS, plasma	6.45	0.001
	Aldehydic derivatives of OMP, plasma	19.61	0.000
	Ketonic derivatives of OMP, plasma	131.33	0.000
	TAC, blood	1.24	0.305

### Localization, age-, and sex-dependent associations of oxidative stress biomarkers

The data presented in Table 4 were analyzed in terms of the localization, sex, and age of all the mute swans studied. The data presented for the analysis were obtained according to the following criteria: biomarkers of lipid peroxidation, aldehydic and ketonic derivatives of oxidatively modified proteins, and total antioxidant capacity in the blood and plasma of mute swans living in the coastal areas of the Pomeranian region, i.e., Słupsk, Gdynia, and Sopot.

**Table 4:** Oxidative stress biomarkers [lipid peroxidation level in blood and plasma (nmol MDA·mL^−1^), aldehydic and ketonic derivatives of oxidatively modified proteins (nmol mL^−1^) and total antioxidant capacity (%)] in the blood and plasma of mute swans of different ages (juveniles and adults) and sexes (males and females) inhabiting three coastal areas of the Pomeranian region (northern Poland)

Age/Sex/ Habitats	Parameters	Mean ± S.D	Age/Sex/ Habitats	Mean ± S.D	Age/Sex/ Habitats	Mean ± S.D
Juveniles, males, Słupsk, n = 10	TBARS, blood	35.20 ± 6.73	Juveniles, males, Gdynia, n = 12	38.20 ± 6.35	Juveniles, males, Sopot, n = 10	31.70 ± 6.08
	TBARS, plasma	11.0 ± 1.47		11.0 ± 1.35		9.10 ± 2.90
	Aldehydic derivatives of OMP, plasma	2.60 ± 0.35		3.0 ± 0.60		6.80 ± 1.96**
	Ketonic derivatives of OMP, plasma	6.0 ± 0.80		4.70 ± 0.76*		12.20 ± 1.75*
	TAC, blood	62.50 ± 8.37		64.8 ± 8.60		52.20 ± 3.42**
Adults, males, Słupsk, n = 12	TBARS, blood	32.20 ± 5.95	Adults, males, Gdynia, n = 18	37.0 ± 6.79	Adults, males, Sopot, n = 17	37.10 ± 6.75
	TBARS, plasma	9.30 ± 1.70		8.30 ± 1.71		3.20 ± 1.66**
	Aldehydic derivatives of OMP, plasma	1.90 ± 1.32		6.60 ± 1.78*		12.40 ± 3.56**^a^
	Ketonic derivatives of OMP, plasma	2.40 ± 0.26^a^		14.0 ± 2.22*^a^		2.50 ± 1.38**^a^
	TAC, blood	44.20 ± 6.14^a^		68.60 ± 3.69*		60.30 ± 10.74^#^
Juveniles, females, Słupsk, n = 10	TBARS, blood	37.20 ± 5.39	Juveniles, females, Gdynia, n = 14	52.0 ± 10.68*	Juveniles, females, Sopot, n = 14	42.40 ± 7.25
	TBARS, plasma	10.80 ± 1.44		7.20 ± 1.31*		9.90 ± 2.11
	Aldehydic derivatives of OMP, plasma	2.40 ± 0.32		3.10 ± 1.56		3.10 ± 0.84
	Ketonic derivatives of OMP, plasma	5.10 ± 0.69		11.10 ± 1.54*^b^		4.0 ± 0.82**
	TAC, blood	22.90 ± 3.09^b^		65.30 ± 8.34*		72.80 ± 10.29^#^
Adults, females, Słupsk, n = 9	TBARS, blood	45.0 ± 8.00		36.60 ± 7.41	Adults, females, Sopot, n = 13	37.20 ± 6.42
	TBARS, plasma	10.90 ± 1.50		11.50 ± 3.30		8.70 ± 1.29^#^
	Aldehydic derivatives of OMP, plasma	11.30 ± 1.50^a^		5.50 ± 2.34*		2.30 ± 1.43**^#^
	Ketonic derivatives of OMP, plasma	14.70 ± 2.0^a^		18.80 ± 3.52* ^a^		4.80 ± 1.07**^#^
	TAC, blood	60.90 ± 8.30^a^		59.60 ± 5.23		67.80 ± 7.91

ANOVA Duncan post hoc tests;

^*^ – statistically significant (p < 0.05) in relation Słupsk–Gdynia;

^**^ – statistically significant (p < 0.05) in relation Sopot–Gdynia;

^#^ – statistically significant (p < 0.05) in relation Słupsk–Sopot;

a – statistically significant (p < 0.05) in relation juveniles–adults;

b – statistically significant (p < 0.05) in relation males–females

We assumed that cell elements contained in blood have their own antioxidant defenses, as convincingly shown in a number of studies (Jóźwik et al., 1997; Kumawat et al. 2012; Fletcher et al. 2005); therefore, we studied the induction of oxidative stress processes in whole blood and plasma separately. Our data showed statistical differences in the baseline levels of TBARS in the blood and plasma of the mute swans studied. The level of lipid peroxidation in the whole blood was three times higher than in the plasma, which suggests a significant role of membrane structures of white and red blood-forming elements and their intracellular processes in the induction and maintenance of antioxidant potential in whole blood. This approach makes it possible to analyze the levels of oxidative stress biomarkers separately in the whole blood and plasma in connection with the three independent parameters presented earlier (localization, age, sex) (Table 4).

When analyzing the group of juvenile birds from the three different habitat locations, we found maximum levels of TBARS in the blood of the mute swans from Gdynia (38.20 ± 6.35 nmol MDA·mL^−1^). At the same time, maximum levels of oxidatively modified protein were found in the swans from Sopot (6.80 ± 1.96 nmol mL^−1^ for aldehydic derivatives of OMP and 12.20 ± 1.75 nmol mL^−1^ for ketonic derivatives of OMP) compared to the mute swans from Słupsk and Gdynia. This trend suggesting high levels of oxidative stress biomarkers was also confirmed by a decrease in the level of total antioxidant capacity (52.20 ± 3.42%) in these groups, which will be discussed below together with other correlation analysis factors (Table 4).

Age-related changes in the initiation of oxidative modification of protein for both forms (aldehydic and ketonic derivatives of OMP) and for one form were shown in the group of the male mute swans living in Słupsk and Sopot. However, these trends were not always detected in the swan representatives from the other localization, which may indicate the influence of other processes, e.g., migration, and other factors.

The statistical analysis revealed age- and sex-dependent differences in the levels of oxidative stress biomarkers, which were observed in the group of the juvenile and adult male and female individuals from Słupsk. The general trend of the changes revealed a significant 44.1% (p < 0.05) reduction of total antioxidant capacity in the blood of adult birds compared to the juvenile ones and a markedly lower total antioxidant capacity in the blood of the juvenile females compared to the adult males (by 60.7%, p < 0.05), respectively. It is possible that the emergence of antioxidant mechanisms in the blood of juvenile birds results from environmental factors and represents a marked adaptation (Table 4).

### Correlative independencies

Determination of the significance of the interaction of the main sex and age effects for the different groups of mute swans required the use of a test of correlative interdependencies to identify the most important relationships (Table 5). These interactions allow a conclusion that the localization in the habitats of mute swans has an impact on lipid peroxidation and the level of oxidatively modified proteins in the blood of the male and female adult swans living in the coastal area of Gdynia and the male adult birds from Sopot. In the present study, we have observed dependencies between the biomarkers of oxidative stress and the Fe, Zn, Al, Rh, and Cu levels. Correlative intergroup interdependency was observed only in the populations from the coastal areas of the Baltic Sea, i.e., Gdynia and Sopot (Table 6).

**Table 5:** Correlative intergroup interdependencies between the levels of oxidative stress biomarkers and metals in the blood and plasma of mute swans of different ages (juveniles and adults) and sexes (males and females) inhabiting three coastal areas of the Pomeranian region (northern Poland)

Age/Sex/ Habitats	Interdependencies	Correlative coefficients	p
Juveniles, Males, Gdynia	TBARS blood – Fe	0.663	0.019
Adults, Males, Gdynia	Aldehydic derivatives of OMP – Fe	-0.471	0.048
Adults, Males, Gdynia	Ketonic derivatives of OMP – Fe	-0.492	0.038
Adults, Females, Gdynia	TBARS plasma – Fe	-0.579	0.049
Adults, Females, Gdynia	Ketonic derivatives of OMP – Fe	0.627	0.029
Adults, Males, Sopot	TBARS plasma – Al	-0.628	0.007
Adults, Males, Sopot	TBARS plasma – Zn	0.624	0.007
Adults, Males, Sopot	TBARS plasma – Rh	0.581	0.014
Adults, Males, Sopot	TBARS plasma – Cu	0.503	0.040
Adults, Males, Sopot	Ketonic derivatives of OMP – Cu	-0.536	0.026
Juveniles, Females, Sopot	TAC – Cu	-0.611	0.020

**Table 6:** SS test for the complete model of the profile of oxidative stress biomarkers (lipid peroxidation level, aldehydic and ketonic derivatives of oxidatively modified proteins, and total antioxidant capacity) and metal levels in mute swans inhabiting three coastal areas of the Pomeranian region (northern Poland)

Parameters	Multiple R	Multiple R^2^	Multiple adjusted R^2^	F	P
TBARS, blood	0.615	0.378	0.329	7.68	0.000
TBARS, plasma	0.604	0.365	0.314	7.25	0.000
Aldehydic derivatives of OMP, plasma	0.863	0.745	0.724	36.88	0.000
Ketonic derivatives of OMP, plasma	0.634	0.402	0.355	8.50	0.000
TAC, blood	0.421	0.177	0.110	2.72	0.003
Al	0.566	0.319	0.266	5.94	0.000
Zn	0.275	0.076	0.003	1.04	0.418
Rh	0.308	0.094	0.023	1.33	0.216
Cu	0.362	0.131	0.062	1.91	0.044
Ru	0.324	0.105	0.035	1.49	0.142
Fe	0.229	0.052	0.023	0.693	0.743
Ni	0.266	0.071	0.003	0.962	0.483

### Complete statistical model of the profile of oxidative stress biomarkers

With the use of multivariable stepwise analysis of statistically significant variables of the main effects (age, sex, and localization), a final model was obtained. The SS test was used to reveal the profiles of biomarkers involved in free radical metabolism interactions and demonstrated the role of these parameters in the formation of the complete model (Table 6). These statistically significant dependencies are as follows: aldehydic derivatives of OMP > ketonic derivatives of OMP > blood TBARS > plasma TBARS > TAC. Statistically significant dependencies in the complete model data were obtained for the Al and Cu levels only, which demonstrated the highest impact of these elements (26.6% for Al and 6.2% for Cu) in the analyzed systems (Table 7).

## Discussion

In the current study, we compared the correlation between the habitat, age, and sex and the accumulation of chemical elements in wintering mute swan populations living in the southern coastal areas of the Baltic Sea (northern Poland). We measured the levels of metals in the soil in three different localities of these mute swan populations and in their feathers. We also analyzed the relationships between metal concentrations and oxidative stress biomarkers in the blood. Finally, we assessed the correlations between the levels of selected metals in both soils collected from the habitats of the mute swans and from their feathers to clarify the alterations in the biomarkers of oxidative stress (lipid peroxidation, oxidatively modified proteins, total antioxidant capacity) (Table 5). Collectively, these results suggest an impact of the habitat, age, and sex separately on the total antioxidant capacity of the birds inhabited from the three coastal areas. These dependencies in bird populations are an important issue for researchers of ecology, physiology, and toxicology, as shown in other studies (Charmantier et al. 2006; Dolka et al. 2014).

There are several new findings in our study. Firstly, the three habitats of the mute swan populations (Słupsk, Gdynia, and Sopot) were characterized by significantly different levels for such metals as Al, Si, Ti, Mn, Fe, Cu, Zn, Zr, Rh, and Ru in the feathers of the mute swans. In Słupsk, the anthropogenic pressure on the waterfowl environment is mainly related to the impact of the Al level and, to a lesser extent, Rh and Ru. In the coastal areas in Gdynia and Sopot, characterized by a high level of anthropogenic pressure associated with recreation and tourism, the predominant impact was exerted by the Rh and Ru levels.

The analysis of the impact of the main effects, i.e., the sex, indicated that the Al and Ru contents in the feathers of the juvenile birds were statistically significantly higher than in the adult group. The Al content was higher in the feathers of the adult mute swans. It is known that aluminum ranks among the most important metals in terms of human use (Gupta et al. 2019). Today, aluminum is one of the elements found in the organism in trace amounts. The main sources of aluminum in the organism are food, water, and atmospheric air (Greger, 1992). Recently, the increasing levels of aluminum and its compounds in the environment, including drinking water sources, have been of particular concern (Léonard and Gerber 1988; Willhite et al. 2014).

Our data on the role of selected elements analyzed in an integrated model showed an important role of element bioaccumulation, except for Al, Rh, and Ru (Table 6). Earlier studies conducted by Kucharska and co-workers (2019) established that blood mercury levels in mute swans from rural breeding sites and urban wintering areas in southern parts of Poland are not related to sex, but are related to age, with no blood parameter implications. These authors showed no difference in Hg concentrations between the sexes, but the Hg concentrations varied significantly between age groups (cygnets, juveniles, and adults). It was concluded that that the Hg concentrations in blood may be influenced by industrialization, season, and age, but generally low concentrations do not affect the hematocrit level and glutathione value (Kucharska et al. 2019).

The current knowledge of the environmental mobility, speciation, and bioavailability with reference to the increased use of platinum-group elements in automobile catalysts has raised concerns about potential environmental and biological accumulation (Hooda et al. 2007). Their role in environmental pollution is related to mechanisms of easy transport thereof to biological materials through deposition in roots by binding to sulfur-rich low molecular weight species in plants. It was noted that palladium and rhodium concentrations have increased in the environment since the introduction of automobile catalysts (Ek et al. 2004). The concentration of these metals has increased significantly in the last decades in diverse environmental matrices, e.g., airborne particulate matter, soil, roadside dust, vegetation, and river, coastal, and oceanic environments (Ravindra et al. 2004). The bioavailability and a greater proportion of these elements in seawater and beaches were associated with emissions from automobile catalysts and roadside surfaces as an end product in samples of road dust, grass, and soil (Wichmann et al. 2007). Importantly, ruthenium is the only platinum-group metal found in living organisms.

Platinum-group elements can be transformed in the soil into more mobile species through complexation with organic matter and can be solubilized in low pH rainwater (Pawlak et al. 2014). Environmentally formed Pd and Rh species are characterized as more soluble and more mobile in the environment. These elements can reach water bodies through stormwater transport and deposition in sediments. Besides external contamination of grass close to roads, internal uptake of platinum-group elements has been observed in plants growing on soil contaminated with these metals by automobile catalysts. Laboratory studies have shown fine particles of platinum-group elements that were detected on the surface of feathers sampled from passerines and raptors in their natural habitat and in internal organs of these birds (Jensen et al. 2002; Ek et al. 2004).

Secondly, our results indicated high-level aluminum-dependent interactions (up to 30%) identified in the mute swan feathers and confirmed in the holistic statistical model (Table 7). The substantially higher Al concentration than that of all other metals analyzed in the bird feathers indicated an impact of this element on the physiological condition and the functional state of free radical production in the blood of the mute swans. The toxicity of Al is related to its competitive interaction with iron in the transferrin molecule and changes in the function of red blood cell membranes by triggering oxidative processes caused by exposure to reactive oxygen species and the disruption or depletion of the cellular antioxidant defense system. Another important target of Al and its compounds is the bone system, where Al forms a complex with citrate and can inhibit the growth of calcium phosphate crystals, which consequently reduces osteoid mineralization (Cannata Andía 1996; Bjørklund et al. 2020). The interest in studying the toxicity of aluminum is caused by its wide distribution in the Earth's crust (8.8%), the variety of its forms in the biosphere, the increasing anthropogenic use recently, and the variety of pathological conditions associated with its excessive intake and further accumulation (Rosseland et al. 1990). Dietary organically complexed aluminum, maybe in synergy with other contaminants, may easily be absorbed and interfere with important metabolic processes in mammals and birds.

Thirdly, our results indicate an association between habitats and the distribution of elements in bird feathers, with involvement of the age and sex of the birds in these processes (Tables 1–4). The highest values of significant differences obtained by the ANOVA test were noted for aldehydic and ketonic derivatives in plasma in the juvenile groups and for aldehydic derivatives of oxidatively modified proteins and total antioxidant capacity in the blood of adults (Table 1). Previous reports have shown that waterfowl are especially sensitive to toxic exposure to xenobiotics in aquatic ecosystems, which have been identified as areas with a high risk of pollution (Birkhead et al. 1982; Perrins et al. 2003; Marchowski et al. 2018). There are many concerns regarding these bird species used in ecological biomonitoring as ideal indicators of pollution caused by various xenobiotics (Ely and Franson 2014; Binkowski et al. 2016). Such results may be related to the constant xenobiotic-induced impact on the organism that may be observed for some metals and species, as shown in the study conducted by Mansouri and Hoshyari (2012). Xenobiotic-induced accumulation in the organisms of waterfowl is determined by their relatively high trophic position in the aquatic food chains, relatively common occurrence, fast-metabolic rates, and long lifespan (Day et al. 2003; Meissner et al. 2020). Many researchers seek an answer to the questions whether the examined areas are contaminated by sufficient levels of heavy metals to cause any adverse effects or poisoning in bird organisms and whether these regions can be considered safe for other wildlife species (Grúz et al. 2018).

Based on the above findings, we estimated the potential impact of the bird sex on the levels of oxidative stress biomarkers. The differences between the levels of the analyzed oxidative stress biomarkers shown by the ANOVA test were statistically significant, except the TBARS and TAC value in the blood of male birds. The levels of aldehydic and ketonic derivatives of OMP in the plasma of the male birds as well as aldehydic derivatives and TAC levels in the female group tended to increase (Table 3).

Based on similar research results, it may be possible to monitor the bioaccumulation of toxic metals in living ecosystems. Since protein sulfhydryl groups in bird feathers readily interact with metals (Jerez et al. 2011), these samples are widely used in research. Feathers of different birds species (Abdullah et al. 2015), and especially mute swan feathers, are effective model systems (Lodenius and Solonen 2013; Grúz et al. 2019). A previous study conducted by Schummer and co-workers (2011) reported an increase in the number of mute swans at Great Lakes despite elevated levels of Cu and Se. This study suggests that these burdens do not substantially limit their reproduction or survival. Such an element as Se was correlated with Cu and Hg levels, which might indicate its interaction in the antioxidant defenses. In our study, statistical interaction between Cu and Fe was noted.

Fourthly, significant differences were observed in the levels such of oxidative stress biomarkers as lipid peroxidation, oxidatively modified proteins, and total antioxidant capacity in the blood of the mute swan population from the coastal areas of Gdynia and Sopot differing in the level and type of anthropogenic pressure (Table 3). It was noted that there were more statistically significant correlations between the oxidative stress markers and the Fe and Cu levels. Recent studies demonstrated the role of these metals in the initiation of lipid peroxidation (Valko et al. 2016). Hydroperoxides (ROOH) and hydrogen peroxide (H_2_O_2_) generated during non-enzymatic lipid oxidation as a product of superoxide radical dismutation in the presence of Fe^2+^, Cu^+^, Co^2+^ ions, i.e., partially reduced metal ions with variable valence, are involved in the generation of additional organic radicals. This ensures the branching of the oxidation chain by forming highly reactive alkoxyl radical RO∙ or hydroxyl radical ∙ON (Mezzaroba et al. 2019).

Free iron in the solution can readily cause oxidation of biological molecules such as lipids and proteins. Therefore, Fe is normally sequestered in vivo by specific binding proteins. If the capacity of these binding proteins is exceeded, oxidative complications may occur (Welch et al. 2002). Cu is necessary for proper mobilization and metabolism of Fe; hence, Cu deficiency may be expected to exacerbate the effects of Fe overload. In Cu deficiency, Fe tends to accumulate in the liver (Harris 1994; Cockell et al. 2005). The levels of transition metal ions in biological media are exceptionally carefully controlled and maintained at extremely low levels of the order of 10^–18^ M. To reduce the likelihood of their involvement in pro-oxidant production, iron and copper ions are retained as part of the biomolecules and are usually in an oxidized state as Fe^3+^ and Cu^2+^ (Dusek et al. 2015). It is supposed that, even in biocomplexes, metal ions with variable valence, after reduction, are still able to catalyze the Haber–Weiss reaction and the formation of the alkoxyl radical. In this case, the role of the reducing agent of transition metal ions can be and usually is performed by the superoxide radical (Filipovic and Koppenol 2019).

In addition, H_2_O_2_-induced and NO-mediated modifications of the structure of copper- and iron-containing proteins also contribute to the labialization of metal ions with variable valence. On the other hand, the loss of iron ions by enzymes involved in energy production and amino acid metabolism is accompanied by a loss of specific activity and their dysfunctions. The appearance of unbound transition metal ions in the environment is a qualitatively new and dangerous state of a biological system (Valko et al. 2016; Andrade et al. 2017). The presence of reduced ions of chemical elements with variable valence is a kind of "reactor" of redox-catabolic production of free radicals, in particular, the extremely toxic hydroxyl radical. Recent studies have shown that many metals occurring separately are also (or mainly) found as mixtures in various parts of the ecosystems, and interactions among the components of the mixture may change the toxicokinetics and toxicodynamics of aquatic and terrestrial coastal ecosystems (Andrade et al. 2017).

## Conclusions

The data presented in the current study regarding the induction of oxidative stress in birds were carried out to determine such oxidative stress biomarkers as lipid peroxidation, aldehydic and ketonic derivatives of oxidatively modified proteins, and total antioxidant capacity in the blood of mute swans inhabiting the coastal areas of Słupsk, Gdynia, and Sopot (Pomeranian region, northern Poland). The levels of chemical elements Al, Si, Ti, Mn, Fe, Cu, Zn, Zr, Rh, and Ru were also measured in bird feathers and soil samples. The interactions between the biomarkers of lipid peroxidation, oxidatively modified proteins, and total antioxidant capacity in the blood of the mute swans and the metal contents in their feathers suggested that there were alterations in their bioaccumulation in the latter material.

The results of the current study indicate an association between the habitat of mute swans and the distribution of metals in their feathers, depending on the age and sex of the birds. The mute swans differed in the Al content in Słupsk, in the Ru value in the coastal area of Gdynia, and in the Ru and Cu levels in Sopot. Based on the multifactor analysis performed in all the studied groups, we estimated the impact of Al at 26.6%, Cu—6.2%, Rh—2.3%, Ru—3.4%, and Fe—2.3% in the whole model. The higher Al levels than those of all the other metals in the feathers of the mute swans indicated the impact of this element on oxidative stress maintenance in the blood of these birds. The highest value of significant differences shown by the ANOVA was noted for aldehydic and ketonic derivatives in the plasma of the juvenile groups and for aldehydic derivatives of oxidatively modified proteins and total antioxidant capacity in the blood of the adults. As we expected, the highest values of significant differences were found for aldehydic and ketonic derivatives of oxidatively modified proteins in the blood of the mute swans from Słupsk and Sopot.

Furthermore, we found that the levels of oxidatively modified proteins were induced by the chemical contamination of the habitats of the mute swans, as shown by the analysis of the soil and feathers. In the current study, we have observed dependencies between the biomarkers of oxidative stress and the Fe, Zn, Al, Rh, and Cu levels. Correlative intergroup interdependencies were observed only in the bird populations from Gdynia and Sopot. The present study demonstrated that the bird populations inhabiting different coastal areas of the southern Baltic Sea were characterized by different metal bioaccumulation resulting in alterations of the levels of biomarkers of oxidative stress in the blood.

## Data Availability

The datasets used and/or analyzed during the current study are available from the corresponding author on reasonable request. All authors have approved the final version of the manuscript and agree to be accountable for all aspects of the work in ensuring that questions related to the accuracy or integrity of any part of the work are appropriately investigated resolved. All persons designated as authors quality for authorship and all those who qualify for authorship are listed.
